# Vildagliptin improves high glucose‐induced endothelial mitochondrial dysfunction via inhibiting mitochondrial fission

**DOI:** 10.1111/jcmm.13975

**Published:** 2018-11-16

**Authors:** Hengdao Liu, Hong Xiang, Shaoli Zhao, Haiqiang Sang, Fenghua Lv, Ruifang Chen, Zhihao Shu, Alex F. Chen, Shuhua Chen, Hongwei Lu

**Affiliations:** ^1^ Center for Experimental Medical Research The Third Xiangya Hospital of Central South University Changsha Hunan China; ^2^ Department of Cardiology The First Affiliated Hospital of Zhengzhou University Zhengzhou Henan China; ^3^ Department of Endocrinology The Third Xiangya Hospital of Central South University Changsha Hunan China; ^4^ Department of Cardiology The First Affiliated Hospital of Xinxiang Medical University Xinxiang Henan China; ^5^ Department of Surgery University of Pittsburgh School of Medicine Pittsburgh Pennsylvania; ^6^ Department of Biochemistry School of Life Sciences Central South University Changsha Hunan China; ^7^ Department of Cardiology The Third Xiangya Hospital of Central South University Changsha Hunan China

**Keywords:** AMPK, Drp1, mitochondrial dysfunction, mitochondrial fission, mitochondrial reactive oxygen species, vildagliptin

## Abstract

The dipeptidyl peptidase 4 inhibitor vildagliptin (VLD), a widely used anti‐diabetic drug, exerts favourable effects on vascular endothelium in diabetes. We determined for the first time the improving effects of VLD on mitochondrial dysfunction in diabetic mice and human umbilical vein endothelial cells (HUVECs) cultured under hyperglycaemic conditions, and further explored the mechanism behind the anti‐diabetic activity. Mitochondrial ROS (mtROS) production was detected by fluorescent microscope and flow cytometry. Mitochondrial DNA damage and ATP synthesis were analysed by real time PCR and ATPlite assay, respectively. Mitochondrial network stained with MitoTracker Red to identify mitochondrial fragmentation was visualized under confocal microscopy. The expression levels of dynamin‐related proteins (Drp1 and Fis1) were determined by immunoblotting. We found that VLD significantly reduced mtROS production and mitochondrial DNA damage, but enhanced ATP synthesis in endothelium under diabetic conditions. Moreover, VLD reduced the expression of Drp1 and Fis1, blocked Drp1 translocation into mitochondria, and blunted mitochondrial fragmentation induced by hyperglycaemia. As a result, mitochondrial dysfunction was alleviated and mitochondrial morphology was restored by VLD. Additionally, VLD promoted the phosphorylation of AMPK and its target acetyl‐CoA carboxylase in the setting of high glucose, and AMPK activation led to a decreased expression and activation of Drp1. In conclusion, VLD improves endothelial mitochondrial dysfunction in diabetes, possibly through inhibiting Drp1‐mediated mitochondrial fission in an AMPK‐dependent manner.

## 1 INTRODUCTION

Chronic vascular complications constitute the main cause of morbidity and mortality in patients with type 2 diabetes (T2DM),^1^ among them endothelial dysfunction represents one of important primary events.^2^ It has been suggested that excess reactive oxygen species (ROS) production and subsequent decrease in vascular bioavailability of nitric oxide (NO) play a causal role in endothelial dysfunction in diabetes.^3^

Mitochondrial dynamics are the integrated processes of mitochondrial fusion, fission, biogenesis, and mitophagy, which are crucial to maintain cellular bioenergetic and redox homeostasis.^4^ Altered mitochondrial dynamics characterized by increased fission but decreased fusion have been demonstrated to be the culprit behind the endothelial dysfunction in diabetes, in which increased mitochondrial fission plays a major role.^2^ Mitochondrial fission is coordinately controlled by dynamin‐related protein‐1 (Drp1) and fission‐1 (Fis1),^5^ and it has been reported that silencing either Fis1 or Drp1 protects high glucose‐induced inhibition of endothelial nitric oxide synthase (eNOS) activity and NO bioavailability, likely by decreasing mitochondrial ROS production.^2^

Growing evidence suggests that dipeptidyl peptidase 4 (DPP‐4) inhibitors, beyond their role in improving glycaemic control, are helpful in ameliorating endothelial dysfunction in humans and animal models of T2DM.^6^, ^7^, ^8^ A recent meta‐analysis supports that DPP4 inhibitors could reduce the risk of adverse cardiovascular events and are safe from a cardiovascular standpoint in patients with T2DM.^9^ The mechanisms of DPP4 inhibitors in this setting are highly associated with decreased inflammation and NADPH oxidase‐related oxidative stress.^8^, ^10^

The major sources of ROS in endothelial cells include mitochondrial electron transport, NADPH oxidase, xanthine oxidase, uncoupled eNOS, and cytochrome P450 enzymes.^11^ It is known that mtROS are biologically important mediators and the concept of mtROS as a driving force for diabetes complications has also been widely accepted.^12^ However, it is unknown the effects of vildagliptin (VLD), an oral DPP‐4 inhibitor, on mtROS production and mitochondrial dysfunction in endothelium. In addition, whether the DPP‐4 inhibitor mediates mitochondrial fission remains unclear.

By using db/db mice, a widely used human T2DM animal model, we found that VLD reduces Drp1 expression and Drp1‐mediated mitochondrial fission in an AMP‐activated protein kinase (AMPK)‐dependent manner. Moreover, inhibition of mitochondrial fission by VLD attenuates diabetes‐induced mitochondrial damage and endothelial dysfunction.

## 2 MATERIALS AND METHODS

### 2.1 Reagents

D‐glucose and mannitol were obtained from Sigma‐Aldrich (St Louis, MO, USA). Primary antibodies against Drp1 (180769), 3‐nitrotyrosine (3‐NT, 61392), 8‐hydroxyguanosine (8‐OHdG, 10802), AMPK (131512), and porin (154856) were from Abcam (Cambridge, MA, USA), and primary antibodies against pAMPK (Thr 172) (2535S), acetyl‐CoA carboxylase (ACC, 3662S), pACC (Ser79) (3661S), eNOS (9586S), peNOS (Ser1177) (9570S), pDrp1(Ser637)(4867S), and GAPDH (2118S) were purchased from Cell Signaling (Beverly, MA, USA). GSK621 (S7898), mdivi‐1 (S7162), and VLD (S3033) were purchased from Selleck Chemicals (Houston, TX, USA). The Drp1 siRNA duplexes and siRNA negative control were designed and synthesized by Ribo‐Bio (Guangzhou, China).

### 2.2 Animal experiments

Eight week‐old male mice C57BLKS/Nju (wildtype, WT) and BKS.Cg‐Dock7 m ^+/+^ Lepr ^db^/Nju (db/db) were obtained from the Nanjing Biomedical Research Institute of Nanjing University. Mice were housed 3‐5 per cage with free access to water and standard chow and under controlled temperature (20 ± 2°C), humidity (60 ± 10%), and lighting (lights on between 6:00 am and 6:00 pm) conditions. After a 1‐week acclimation period, mice were randomly allocated to five groups including one nondiabetic WT normal group and four diabetic groups, six mice each, with matched bodyweight and blood glucose levels. Diabetic mice were given drinking water (diabetic control group), VLD (orally, 35 mg/kg dissolved in drinking water, once per day, dbVLD group), mdivi‐1 (intraperitoneally, 10 mg/kg dissolved in dimethyl sulfoxide (DMSO), twice per week, dbMDI group), or DMSO (intraperitoneally, 10 mg/kg, twice per week, dbDMSO group) for 6 weeks based on previous studies.^13^, ^14^, ^15^ At the end of treatments, aortas and serum were collected and total cholesterol and triglyceride levels in serum were measured. All animal experiments were performed with approval from the Animal Ethics Committee of the Third Xiangya Hospital of Central South University (LLSC (LA) 2017‐002).

### 2.3 Cell culture

Human umbilical vein endothelial cells (HUVECs) purchased from American Type Culture Collection (Manassas, VA, USA) were grown in DMEM supplemented with 10% FBS at 37°C in a 5% CO_2_ humidified atmosphere. Cells were treated with 5.6 mM glucose (normal glucose group, normal group), 5.6 mM glucose plus 25 mM mannitol (Man group, osmotic pressure control), 30 mM glucose (high glucose group, HG group), or 30 mM glucose plus 1.0 μM VLD (HG+VLD group) for 48 h as previously described.^16^, ^17^, ^18^ Besides, to confirm the requirement of AMPK activation for Drp1 expression, a selective AMPK agonist GSK621 (10 μM) was used as previously described.^19^, ^20^

### 2.4 Transfection with small interfering RNA (siRNA)

To verify the function of Drp1 in HUVECs, Drp1 siRNA was delivered into cells according to the manufacturer's instructions. Briefly, cells were transfected with 20 μM Drp1 siRNA or negative control siRNA using Lipofectamine 2000 reagent (Invitrogen, Carlsbad, CA, USA) for 48 h, and then stimulated with high glucose for another 48 h.

### 2.5 Assessment of ROS and mtROS production in vascular and endothelial cells

Vascular ROS and mtROS productions were determined using 2,7‐dichlorodihydro‐fluorescein diacetate (DCF‐DA, Beyotime, Shanghai, China) and MitoSox Red (Thermofisher Scientific, Waltham, MA, USA) as described previously.^21^, ^22^ Briefly, freshly isolated aortic rings were incubated with 10 μM DCF‐DA or 5 μM MitoSox Red in Hank's buffered salt solution (HBSS) at 37°C for 1 h, and then cut into arterial sections at a thickness of 10 μm using a cryostat microtome and finally mounted serially on slides. Following 4′,6‐diamidino‐2‐phenylindole (DAPI) staining and washing twice in HBSS, fluorescent images were captured with an Olympus fluorescent microscope. For intracellular ROS production, HUVECs were incubated with 10 μM DCFH‐DA and 5 μM MitoSox for 30 min at 37°C. Fluorescent images were captured after washing cells twice. The fluorescence intensity was quantified using Image J software (National Institutes of Health, Bethesda, MD, USA). At the same time, treated cells were collected and resuspended in 200 μl of PBS buffer for flow cytometric analysis of ROS and mtROS.

### 2.6 Immunohistochemistry assay

Immunohistochemical staining was performed to detect 8‐OHdG and 3‐NT in endothelial cells in mouse aortas as described previously.^14^ The numbers of 3‐NT‐ and 8‐OHdG‐positive cells were counted and normalized to the total numbers of endothelial cells in the same field with the use of computer‐assisted morphometric analysis.

### 2.7 Measurement of NO production

NO production in culture medium and serum was determined by quantitation of stable metabolites of NO, nitrite and nitrate, using a commercial Nitric Oxide Assay kit (Nanjing Jiancheng Bioengineering Techology, Nanjing, China).

### 2.8 Mitochondrial DNA damage analysis

Mitochondrial DNA (mtDNA) damage in HUVECs was analysed by quantitative PCR as previously described.^23^ Briefly, DNA was extracted using a QIAamp DNA Micro kit (Qiagen, West Sussex, UK) and real‐time PCR reactions were performed on the Mastercycler EP realplex PCR System (Eppendorf, Wesseling‐Berzdorf, Germany) according to manufacturer's protocol. The primers for mtDNA and β‐globin were as follows: mtDNA (sense: 5′‐CCC CAC AAA CCC CAT TAC TAA ACC CA‐3′; antisense: 5′‐TTT CAT CAT GCG GAG ATG TTG GAT GG‐3′); β‐globin (sense: 5′‐CGA GTA AGA GAC CAT TGT GGC AG‐3′; antisense: 5′‐GCT GTT CTG TCA ATA AAT TTC CTTC‐3′). Gene expression was calculated by using the comparative cycle threshold (ΔΔ Ct) method and relative expression of mtDNA was normalized with β‐globin.

### 2.9 ATP synthesis detection

Cells were seeded into 96‐well plates in triplicate and treated with indicated compounds for indicated time, and finally collected for ATP detection using an ATPlite assay (Perkin‐Elmer, Waltham, MA, USA).

### 2.10 Mitochondrial morphology analysis

To observe the morphological changes of mitochondria, cells were stained with MitoTracker Red (100 nM; Molecular Probes, ThermoFisher Scientific) at 37°C for 30 min, captured by LSM800 confocal microscopy (Carl Zeiss, Jena, Germany), and analysed using Image J software. Mitochondria were subjected to ‘analyse particles’ to obtain mitochondrial interconnectivity (ratio of area and perimeter) and mitochondrial elongation (ratio of the lengths of major and minor axes), two well‐characterized mediators of mitochondrial fission and fusion as described before.^24^ At least 100 cells were analysed in each sample to determine cells undergoing mitochondrial fragmentation.

### 2.11 Western blotting analysis

Western blotting was performed as described previously.^16^ Samples were separated by 10% SDS‐PAGE and then transferred to PVDF membrane (Millipore, Billerica, MA, USA). After blocking with 5% nonfat dried milk, the membranes were incubated with primary antibodies at 4°C overnight and then incubated with goat anti‐rabbit IR‐Dye 800cw labelled secondary antisera in LiCor blocking buffer at room temperature for 1 h. Membranes were imaged using a LiCor Odyssey scanner (LI‐COR, Lincoln, NE).

### 2.12 Statistical analysis

All statistical analyses were performed using SPSS 18.0 software (SPSS Inc., Chicago, IL, USA). All experiments were repeated at least thrice. Data are presented as mean ± standard deviation (SD) for continuous variables and as numbers with percentages for categorical variables. Differences between groups were analysed by one‐way ANOVA followed by the post‐hoc Tukey's test for continuous variables and by χ^2^ tests for categorical variables. A value of *P < *0.05 was considered significant.

## 3 RESULTS

### 3.1 The effects of VLD on metabolic parameters

Following 6‐week VLD treatment in animals, blood glucose, bodyweight, triglyceride, and total cholesterol were measured. The levels of these physical and biochemical parameters were all increased in the diabetic control mice when compared to the nondiabetic WT mice. However, serum triglyceride concentration and blood glucose level were significantly decreased in the VLD group compared to the diabetic control group. Upon VLD treatment, body weight and total cholesterol declined, but no statistical differences were seen between the mice treated with VLD and the mice in the diabetic control group (Table 1).

**Table 1:** The effects of VLD on metabolic parameters

	BG (mM)	BW (g)	TG (mM)	TC (mM)
Normal	8.72 ± 1.89	24.53 ± 0.84	0.86 ± 0.17	1.15 ± 0.12
db CON	29.68 ± 1.91*	41.95 ± 0.97*	1.52 ± 0.23*	1.96 ± 0.16*
db VLD	21.97 ± 2.90*#	40.78 ± 1.33	1.25 ± 0.20*#	1.78 ± 0.19*

Following 6‐week VLD treatment, blood glucose, body weight, triglyceride, and total cholesterol of animals were measured immediately. Data shown are mean ± SD (n = 6). *P <* *0.05 vs the normal group; ^#^ *P *<* *0.05 vs the diabetic control group. BG, blood glucose; BW, body weight; TG, triglyceride; TC, total cholesterol.

### 3.2 VLD reduces ROS, especially mtROS, in endothelium under diabetic conditions

**Figure 1:**
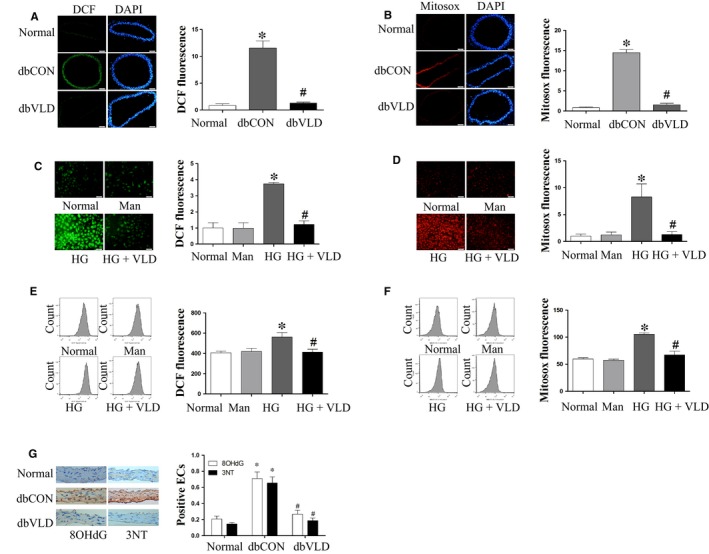
Vildagliptin (VLD) alleviates ROS, especially mtROS in the endothelium under diabetic conditions. A‐B, Effect of VLD on vascular ROS and mtROS in mice. Freshly isolated aortic rings were incubated with 10 μM DCF‐DA or 5 μM MitoSox Red at 37°C for 1 h. After cutting into segments with the thickness of 10 μm and staining with DAPI, images were captured by a fluorescent microscope at ×200 magnification. C‐F, Effect of VLD on ROS and mtROS in HUVECs. Cells were incubated with 10 μM DCF‐DA or 5 μM MitoSox Red for 30 min at 37°C and then captured using a fluorescent microscope or analysed by flow cytometry. G, Effect of VLD on 8‐OHdG and 3‐NT levels in mice aortas detected by immunohistochemical staining. Data shown are mean ± SD (n = 3‐6). *P<0.05 vs the normal group; ^*#*^ *P *<* *0.05 vs the HG group or the diabetic control group. VLD, vildagliptin; ROS, reactive oxygen species; mtROS, mitochondrial reactive oxygen species; HUVECs, human umbilical vein endothelial cells; HG, high glucose; Man, mannitol

In comparison to the nondiabetic WT mice, the diabetic control mice exhibited significantly increased production of ROS and mtROS in aortic endothelium. However, VLD treatment returned the production of ROS and mtROS in the diabetic mice to the baseline levels of the nondiabetic WT group (Figure 1A,B). Interestingly, similar inhibitory effects of VLD on ROS and mtROS production were achieved in vitro in HUVECs in the presence of high glucose. There were no significant differences in the production of ROS and mtROS in HUVECs between the osmotic control group and the normal group (Figure 1C‐F).

We further explored the suppressive effects of VLD on diabetes‐induced oxidative stress by detecting the levels of 8‐OHdG (a biomarker for oxidative DNA damage) and 3‐NT (a marker of oxidative damage by protein tyrosine nitration) in the db/db mouse aortas. As expected, VLD significantly inhibited hyperglycaemia‐induced increases in the levels of 8‐OHdG and 3‐NT (Figure 1G). Collectively, these results suggest that VLD has the capacity to inhibit ROS production and oxidative damage in endothelium.

### 3.3 VLD promotes NO production in endothelium under diabetic conditions

**Figure 2:**
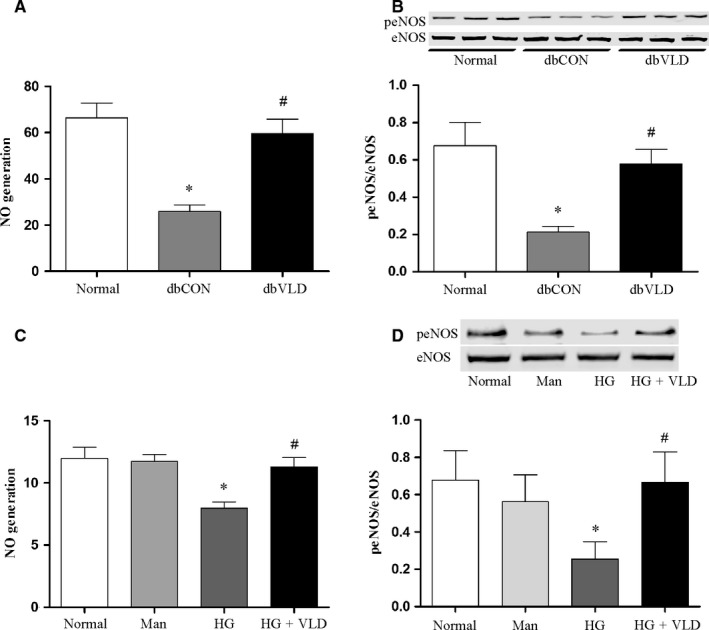
VLD promotes NO generation and expression of peNOS in the endothelium under diabetic conditions. Serum or cell supernatant was collected and total NO production was measured by a modified Griess reaction method. The activation of eNOS was evaluated by assaying eNOS phosphorylation by Western blot. A‐B, Effects of VLD on NO production and eNOS activation in the aortas of mice. C‐D, Effect of VLD on NO production and eNOS activation in HUVECs. Data shown are mean ± SD (n = 3‐6). *P < 0.05 vs the normal group; ^*#*^ *P < *0.05 vs the HG group or the diabetic control group. Cell supernatant was collected for total NO production. VLD, vildagliptin; NO, nitric oxide; eNOS, endothelial nitric oxide synthase; peNOS, phosphorylation of eNOS at Ser1177; HUVECs, human umbilical vein endothelial cells; HG, high glucose; Man, mannitol

In comparison to the nondiabetic WT mice, NO production was significantly decreased in the diabetic control mice (Figure 2A). Given that the phosphorylation of eNOS at Ser1177 is a critical regulatory process for NO generation,^25^ the phosphorylation status of eNOS was therefore evaluated herein. As expected, the phosphorylation levels of eNOS were significantly reduced in the diabetic control group (Figure 2B), consistent with the altered NO production. However, VLD blunted diabetes‐induced decreases in eNOS activation and NO production (Figure 2A,B). Similarly, the effects of VLD on eNOS activation and its product NO were observed in HUVECs under high glucose conditions. No significant changes in NO production and peNOS levels in HUVECs were observed between the osmotic control group and the normal group (Figure 2C,D).

### 3.4 VLD improves mitochondrial dysfunction and mitochondrial fragmentation induced by high glucose in endothelial cells

**Figure 3:**
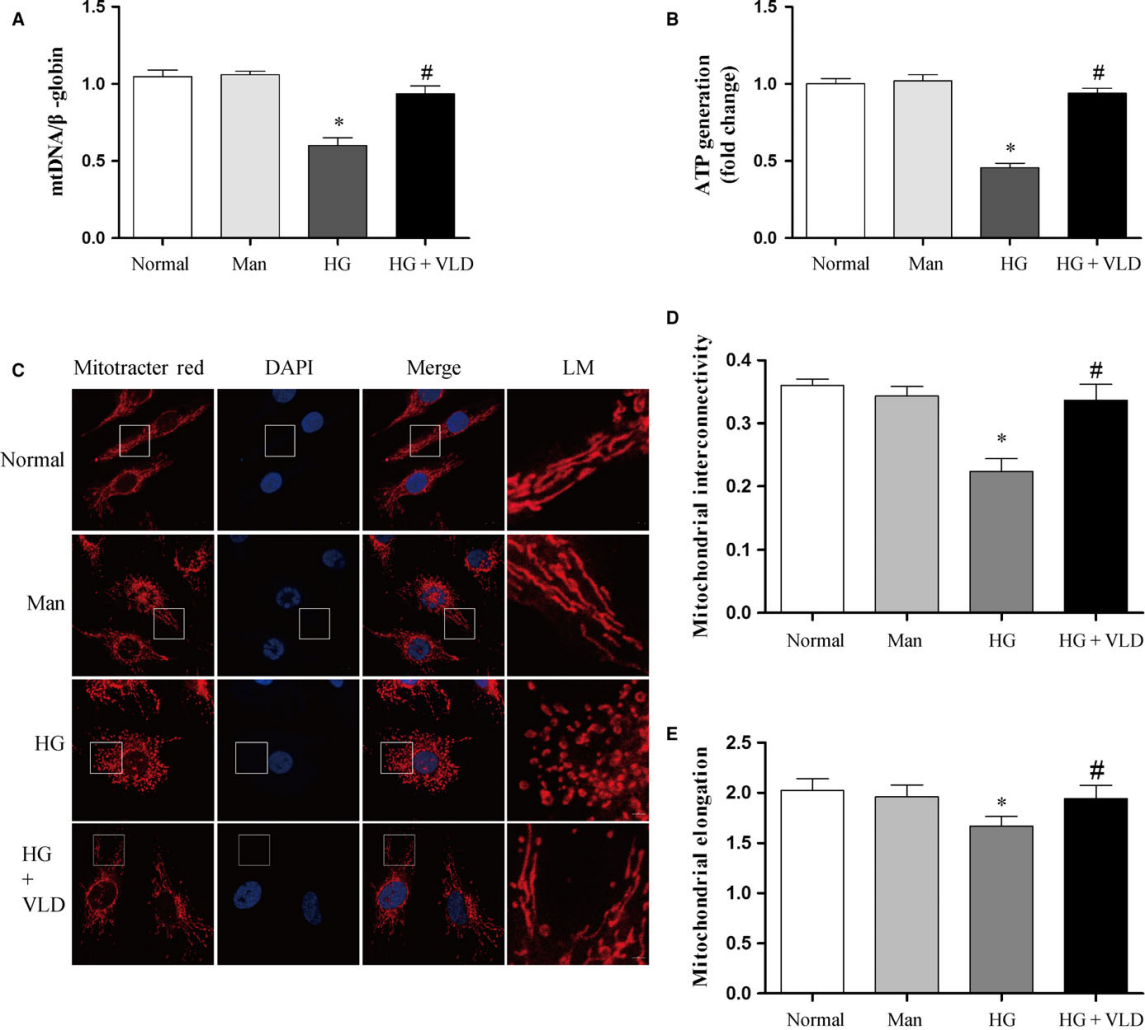
VLD improves mitochondrial dysfunction and mitochondrial fragmentation induced by high glucose in endothelial cells. A, Effect of VLD on mtDNA damage in HUVECs under hyperglycemic conditions. Total DNA was extracted and mitochondrial DNA damage determined by quantitative PCR in HUVECs. B, Effect of VLD on ATP production in HUVECs in response to hyperglycemia. ATP detection was performed by an ATPlite assay. C, Effect of VLD on mitochondrial morphology in HUVECs. Cells were stained with MitoTracker Red for 20 min at 37°C and images captured using a confocal microscopy. D‐E, Quantitative image analysis of mitochondrial interconnectivity and mitochondrial elongation by Image J. Data shown are mean ± SD (n = 3). ^*^ *P < *0.05 vs the normal group; ^*#*^ *P < *0.05 vs the HG group. VLD, vildagliptin; mtDNA, mitochondrial DNA; HUVECs, human umbilical vein endothelial cells; HG, high glucose; Man, mannitol; LM, local magnification

Since mitochondrial oxidative stress leads to mtDNA damage and lower mtDNA content usually indicates more serious DNA damage, mtDNA content was then assessed in vitro. We observed that in the presence of high glucose, mtDNA content was significantly reduced in HUVECs. However, VLD treatment significantly increased mtDNA content compared to the high glucose group (Figure 3A). Besides, high glucose alone induced a significance decrease in ATP production compared to the normal glucose group. However, pretreatment of HUVECs with VLD significantly inhibited high glucose‐induced change in ATP production (Figure 3B). The mtDNA content and ATP production in the osmotic control with 30 mM mannitol remained unchanged compared to those of the normal glucose group. These results suggest that VLD provides a protective effect against oxidative stress‐mediated mitochondrial dysfunction.

We further explored the effects of VLD on mitochondrial network fragmentation in endothelial cells. Mitochondrial network of HUVECs in the normal glucose and mannitol groups was interconnected and extensive throughout the cells. However, the mitochondrial network of cells in the high glucose group was significantly disturbed and scattered (Figure 3C), and average mitochondrial interconnectivity and elongation values were significantly decreased, suggestive of mitochondrial fragmentation.^24^ Interestingly, administration of VLD reduced diabetes‐induced mitochondrial fragmentation compared to the high glucose group (Figure 3D,E).

### 3.5 VLD inhibits high glucose‐induced mitochondrial fission by regulation of dynamin‐related proteins

**Figure 4:**
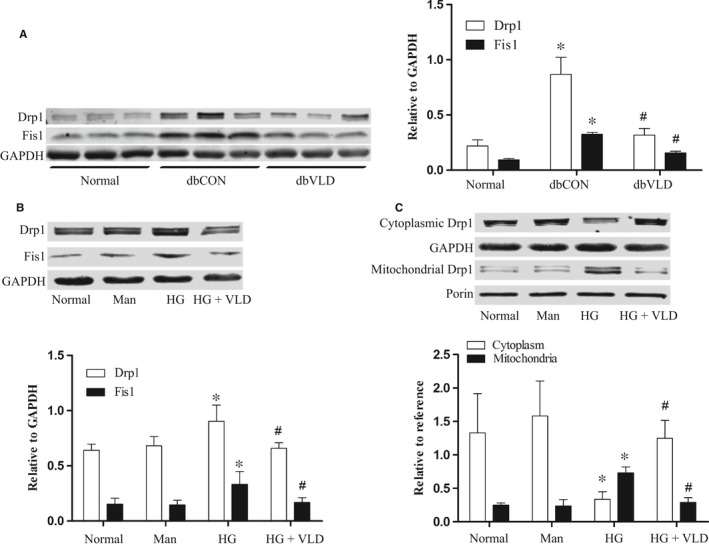
VLD inhibits high glucose‐induced mitochondrial fission by regulation of dynamin‐related proteins. A, Effects of VLD on the expression of Drp1 and Fis1 in mice aortas. B, Effect of vildagliptin on the expression of Drp1 and Fis1 in HUVECs. C, Effect of VLD on Drp1 recruitment from cytoplasm to mitochondria. Data shown are mean ± SD (n = 3‐6). *P<0.05 vs the normal group; ^*#*^ *P *<* *0.05 vs the HG group or the diabetic control group. VLD, vildagliptin; HUVECs, human umbilical vein endothelial cells; HG, high glucose; Man, mannitol

Now that Drp1 and Fis1 are essential regulators of mitochondrial fission, we tested their protein expression changes following VLD treatment. Compared with the nondiabetic WT mice aortas, aortas of the diabetic control mice exhibited significantly increased expression of Drp1 and Fis1, but VLD treatment obviously reduced the Drp1 and Fis1 expression (Figure 4A). It is also true for VLD effect in high glucose‐treated HUVECs, that is, VLD significantly inhibited high glucose‐induced up‐regulation of Drp1 and Fis1 under hyperglycaemia conditions. However, VLD did not affect the expression of Drp1 and Fis1 in the normal glucose treated HUVECs (Figure S1A), and there were no significant changes of Drp1 and Fis1 expression in HUVECs between the osmotic control group and the normal group (Figure 4B). In addition, we observed VLD effectively reduced Drp1 recruitment to mitochondria (Figure 4C)**.**

Moreover, we examined the effects of VLD on the expression of mitochondrial fusion proteins under high glucose conditions. We found no difference in the protein levels of MFN1 (*P = *0.753) and MFN2 (*P = *0.852) between all groups, while OPA1 exhibited a nonsignificant trend to decrease in the high glucose group compared with the normal group(*P = *0.068) but increase in the high glucose plus group compared with the high glucose group (*P = *0.064) (Figure S2).

### 3.6 VLD inhibits Drp1 activation via regulation of AMPK activation

**Figure 5:**
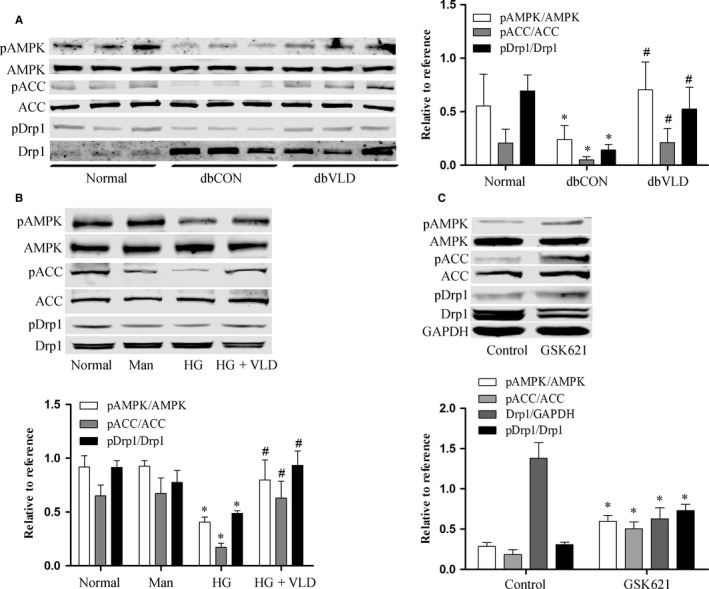
VLD inhibits Drp1 activation via the regulation of AMPK activation. A‐B, Effects of VLD on the phosphorylation of AMPK, ACC and Drp1 in mice and in HUVECs. The levels of pAMPK, pACC and pDrp1 were evaluated by Western blot. C, AMPK activation was essential for Drp1 expression and activation in HUVECs. The phosphorylation levels of AMPK and ACC as well as Drp1 and pDrp1 expression in HUVECs were evaluated by Western blot following cell exposure to GSK621, a specific AMPK activator. Data shown represent mean ± SD (n = 3‐6). *P<0.05 vs the normal group; ^*#*^ *P < *0.05 vs the HG group or the diabetic control group. VLD, vildagliptin; HUVECs, human umbilical vein endothelial cells; HG, high glucose; Man, mannitol; pAMPK, phosphorylation of AMPK at Thr172; pACC, phosphorylation of ACC at Ser79; pDrp1, phosphorylation of Drp1 at Ser637

AMPK activation is required for mitochondrial fragmentation,^14^ we therefore tested the phosphorylation of AMPK at Thr172 and phosphorylation of acetyl‐CoA carboxylase (ACC) at Ser79 as a surrogate indicator of enzyme activity. High glucose and diabetes inhibited the phosphorylation of AMPK and its downstream target ACC in HUVECs and mouse aortas, respectively, but the decreased phosphorylation levels of AMPK and ACC by high glucose in vitro and diabetes in vivo were reversed by VLD treatment (Figure 5A,B). Besides, Drp1 phosphorylation at the residue of Ser637 has been demonstrated to inactivate Drp1, preventing its translocation to mitochondria. We found that high glucose also decreased S637 phosphorylation, which was, however, improved by VLD. Moreover, VLD did not affect the expression of pAMPK and pACC and the phosphorylation of Drp1 at Ser637 compared to those in the normal glucose‐treated HUVECs (Figure S1B). Furthermore, we tested whether AMPK activation is essential for Drp1 expression and S637 phosphorylation in HUVECs. AMPK (Thr172) activation by GSK621 resulted in increased pACC expression, decreased Drp1 expression and increased S637 phosphorylation, indicative of the regulation of Drp1 by AMPK (Figure 5C).

### 3.7 Inhibition of Drp1 attenuates production of ROS and mtROS and improves mitochondrial fragmentation and dysfunction under high glucose condition

**Figure 6:**
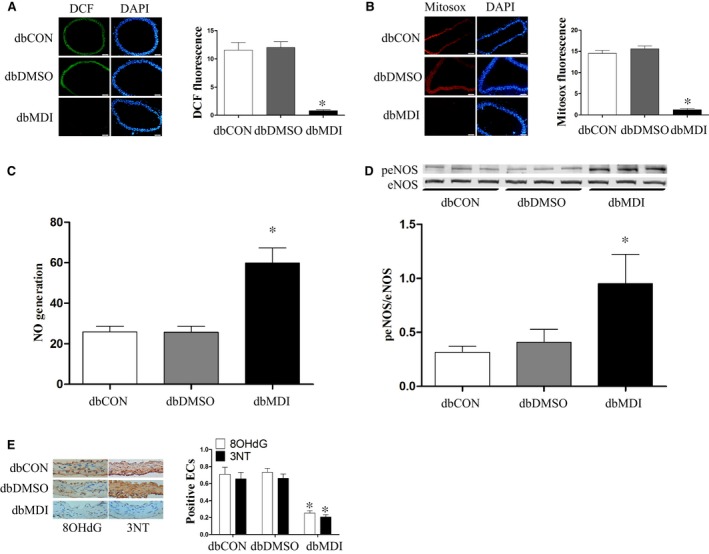
Inhibition of Drp1 attenuates ROS and mtROS production and increases NO generation in diabetic mice. MtROS, ROS, and NO production in vascular were determined as described in Figures 1 and 2. A‐D, Effect of mdivi‐1 on vascular mtROS, ROS, and NO production in mice. E, Effects of Drp1 knockdown on the levels of 8‐OHdG and 3‐NT in mice aortas. Data shown represent mean ± SD (n = 6). *P < 0.05 vs the diabetic control group. ROS, reactive oxygen species; mtROS, mitochondrial reactive oxygen species; NO, nitric oxide; eNOS, endothelial nitric oxide synthase; peNOS, phosphorylation of eNOS at Ser1177

Compared with the diabetic control group, the mdivi‐1 group displayed significantly decreased ROS and mtROS production but obviously increased NO generation and eNOS phosphorylation in aortic endothelium (Figure 6A‐D). As a result, the levels of 8‐OHdG and 3‐NT as indicators of macromolecular oxidation were significantly reduced in the db/db mouse aortas (Figure 6E).

**Figure 7:**
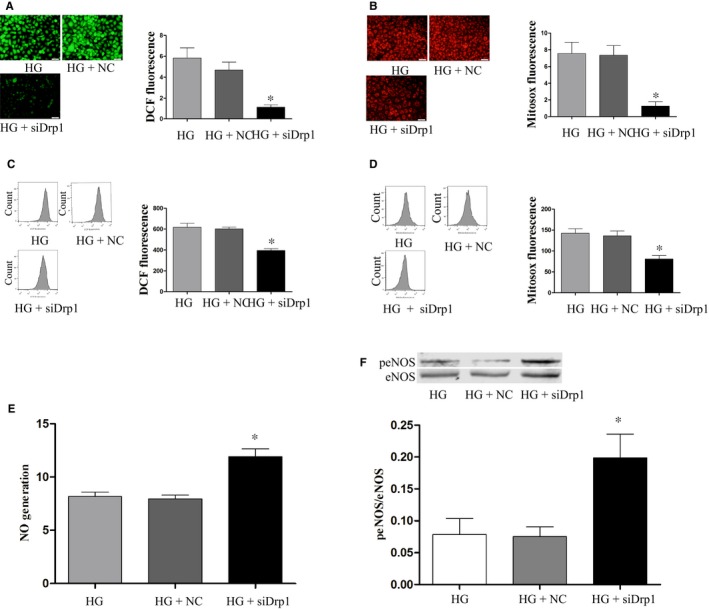
Drp1 knockdown attenuates ROS and mtROS production and increases NO generation in HG‐treated HUVECs. MtROS, ROS and NO production in HUVECs were determined as described in Figures 1 and 2. Data shown represent mean ± SD (n = 3). *P < 0.05 vs the HG group. ROS, reactive oxygen species; mtROS, mitochondrial reactive oxygen species; HUVECs, human umbilical vein endothelial cells; NO, nitric oxide; eNOS, endothelial nitric oxide synthase; peNOS, phosphorylation of eNOS at Ser1177; HG, high glucose; NC, negative control

**Figure 8:**
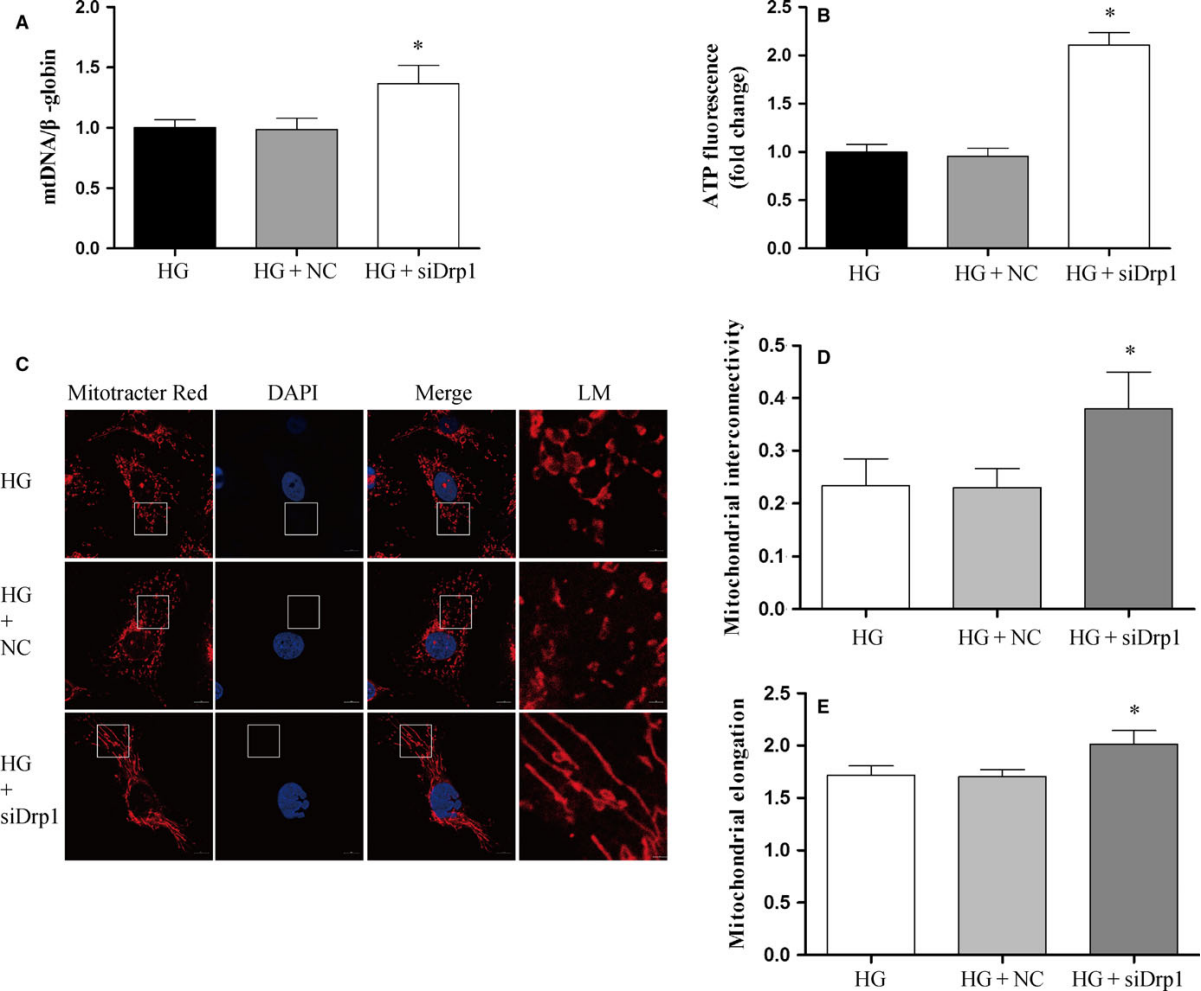
Inhibition of Drp1 improves mitochondrial dysfunction and mitochondrial fragmentation induced by high glucose in HUVECs. A, Effects of Drp1 knockdown on mtDNA damage in HUVECs under hyperglycemic conditions. Total DNA was extracted and mitochondrial DNA damage was evaluated by quantitative PCR in HUVECs. B, Effects of Drp1 knockdown on ATP production in HUVECs under hyperglycaemic conditions. ATP detection was performed by an ATPlite assay. C, Effects of Drp1 knockdown on mitochondrial morphology in HUVECs. Mitochondrial staining and imaging were performed as described in Figure 4. D‐E, Quantitative image analysis of mitochondrial interconnectivity and mitochondrial elongation by Image J. Data shown are mean ± SD (n = 3), *P <* *0.05 vs the HG group. mtDNA, mitochondrial DNA; HUVECs, human umbilical vein endothelial cells; HG, high glucose; NC, negative control; LM, local magnification

Similarly, the varying amounts of produced ROS, mtROS, and NO as well as eNOS phosphorylation were seen in HUVECs under high glucose conditions once Drp1 was knocked down (Figure 7A‐F). Additionally, under high glucose conditions mtDNA content and ATP production were significantly reduced while mitochondrial fragmentation was enhanced, but Drp1 knockdown effectively reversed these phenomena, suggesting the involvement of Drp1 in oxidative stress response and mitochondrial fragmentation and dysfunction (Figure 8A‐E).

### 3.8 Effects of VLD on the expression of ROCK1 and BNIP3 in HUVECs under high glucose conditions

ROCK1 has been demonstrated to participate in Drp1 mediated‐mitochondrial fission under hyperglycaemia conditions.^26^ In the present study, we further confirmed that high glucose promotes the protein expression of ROCK1 compared with the normal group (*P < *0.001), and VLD inhibited that of ROCK1 compared with high glucose group though no significant effects were found (*P = *0.107) (Figure S3A). Moreover, previous studies suggested that BNIP3 mediates Drp1 activation in cardiac myocytes,^27^ however, we did not find a significant change in the protein expression of BNIP3 in HUVECs under high glucose condition (Figure S3B), in agreement with another study using hyperglycaemia‐induced cardiac myocytes.^28^

## 4 DISCUSSION

In the present study, we demonstrated for the first time that VLD improves high glucose‐induced endothelial dysfunction by alleviating mitochondria oxidative stress via the regulation of mitochondrial fission. In both diabetic db/db mice and hyperglycaemia‐exposed HUVECs, we confirmed that VLD reduces Drp1 and Fis1 expression and subsequently mitochondrial fission mediated via an AMPK‐dependent manner. The inhibition of mitochondrial fission by VLD leads to a decline in mitochondria oxidative stress and mitochondrial dysfunction, and the improvement of endothelial function, which is associated with the increased availability of NO via eNOS activation.

Emerging evidence suggests that DPP‐4 inhibitors, including VLD, produce favourable effects on the vascular endothelium in diabetes.^29^ Specifically, DPP‐4 inhibitors stimulate NO production by enhancing eNOS activity, inhibit endothelium‐derived vasoconstrictory factors such as endothelin‐1,^29^ and decrease the serum levels of plasminogen activator inhibitor‐1 and matrix metallopeptidase 9.^30^, ^31^ Besides, DPP‐4 inhibitors improve endothelial function by increasing circulating endothelial progenitor cells in T2DM patients with concomitant up‐regulation of stromal cell‐derived factor‐1α.^32^ Furthermore, excessive production of ROS and subsequent decrease in NO production result in endothelial dysfunction. Previous studies have shown that teneligliptin reduces vascular oxidative stress in patients with T2DM.^33^

As a by‐product of mitochondrial oxidative phosphorylation, mtROS are a predominant type of ROS, and overproduction of mtROS contributes to the dysfunction of endothelial cells in diabetic vascular complications.^34^, ^35^ In the current study, administration of VLD was demonstrated to decrease the increment of mtROS, and subsequently reverse the decreased NO production induced by hyperglycaemia, which was mediated by upregulated phosphorylation of eNOS at Ser1177. Since mitochondria are a primary target for the destructive actions of mtROS,^11^ which leads to mitochondrial damage, including a decreased mitochondrial ATP synthesis, and induction of mtDNA damage. Our data support that VLD inhibits mtDNA damage and enhances ATP production under high glucose conditions.

Mitochondria are remarkably dynamic organelles for the sustained balance of fission and fusion.^36^ Accumulated data suggest that mitochondrial fission characterized by obvious increment of mitochondrial fragmentation is enhanced in diabetic status.^2^, ^14^ In the presence of high glucose the expression of Drp1 and Fis1 may remain stably while fusion‐related proteins such as mitofusin‐2 and optic atrophy type 1 do not change significantly.^2^ In our study, VLD was observed not only to inhibit overexpression of both Drp1 and its downstream protein, Fis1, but to improve the downexpression of Ser637 phosphorylation of Drp1 induced by high glucose, thus further resulting in a decreasing Drp1 activity that limits its intracellular location primarily in the cytosol.^37^ Collectively, these changes attribute to the declined mitochondria fission and fragment caused by hyperglycaemia. Furthermore, by using the Drp1 inhibitor mdivi‐1 in the diabetic mice and Drp1siRNA in the high glucose‐treated HUVECs, we validated that a reduced mitochondrial fission will improve mitochondrial damage induced by hyperglycaemia.

AMPK is a key regulator of energy balance and a central metabolic sensor activated by a wide variety of mitochondrial insults.^38^, ^39^ Previous studies have shown that AMPK activation was sufficient to regulate mitochondrial fission by reducing Drp1 expression and inhibiting its activity.^14^, ^40^ By using the AMPK activator GSK621, we confirmed that the phosphorylation of AMPK enhanced pACC expression, resulting in the reduction in Drp1 expression and Ser637 phosphorylation in HUVECs. Furthermore, the phosphorylation levels of AMPK and ACC was decreased in the diabetic mouse aortas and HUVECs under high glucose conditions, in line with previous studies.^14^ However, the reduced phosphorylation of AMPK and ACC was prevented by VLD, similar to the effect of another DPP4 inhibitor teneligliptin, which attenuates hepatic lipogenesis by activating AMPK.^41^ In all, these results suggest that VLD reduces Drp1 expression and activation via the promotion of AMPK phosphorylation.

Administration of VLD improved lipid profile in the present study, in agreement with previous reports.^42^, ^43^ Besides, VLD treatment reduced the level of blood glucose in the diabetic mice, which may further prevent endothelial dysfunction. However, the hypoglycemic effect of DPP‐4 inhibitors is achieved by increasing the incretin hormone, predominantly glucagon‐like peptide 1 and glucose‐dependent insulinotropic polypeptide,^6^ and it is therefore hard to come to a conclusion to claim who plays a leading role in the diabetic mice. However, because of the absence of incretin hormone in HUVECs, the protection of DPP4 inhibitors in the endothelium may be independent of hypoglycaemia.

In conclusion, VLD represses mitochondrial ROS and improves mitochondrial dysfunction through inhibiting mitochondrial fission in diabetes. In addition, AMPK activation by VLD decreases Drp1‐mediated mitochondrial fragmentation under hyperglycaemic conditions.

## CONFLICTS OF INTEREST

None.
