# Supplementary figures and images for: Vildagliptin improves high glucose‐induced endothelial mitochondrial dysfunction via inhibiting mitochondrial fission
Source: J Cell Mol Med. 2018 Nov 16;23(2):798–810. doi: 10.1111/jcmm.13975 (PMC6349192; doi:10.1111/jcmm.13975)

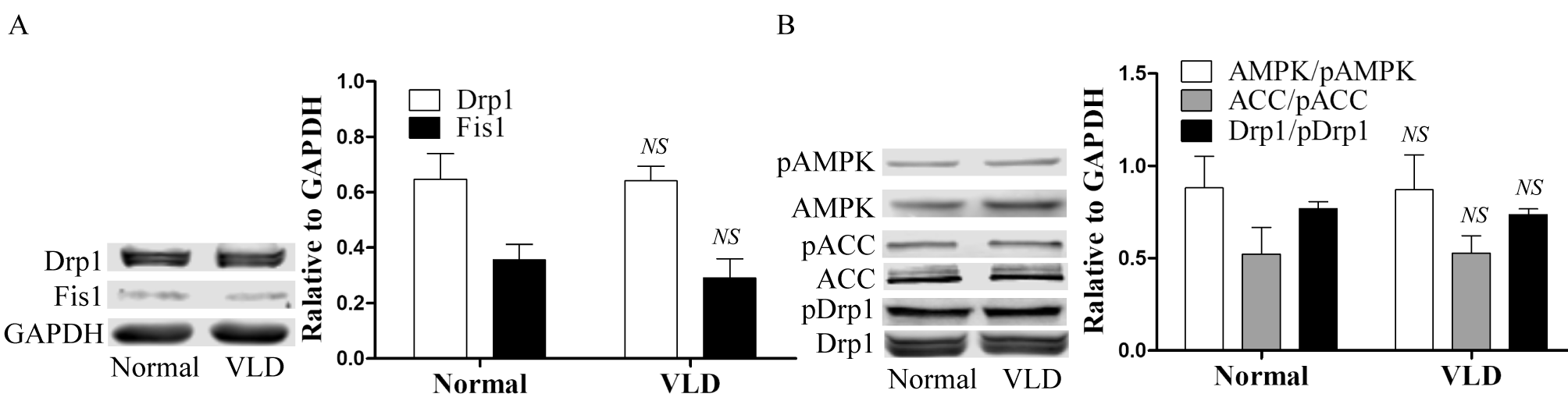

Supplement: Supplementary file 1 [file JCMM-23-798-s001.tif]

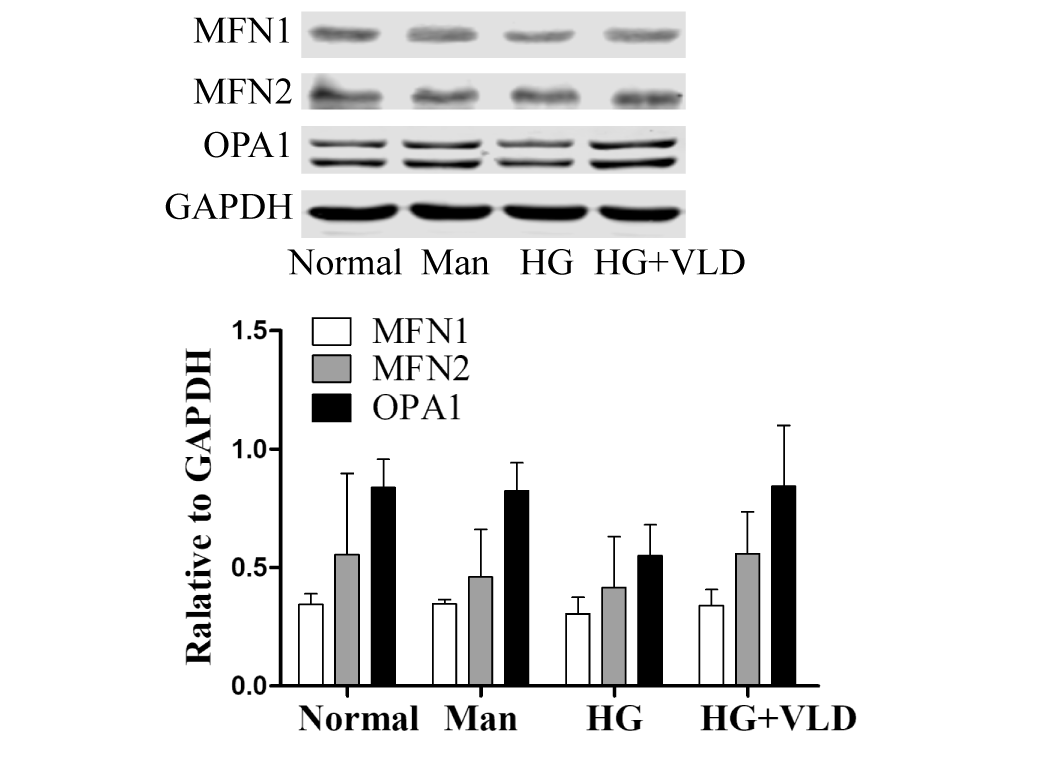

Supplement: Supplementary file 2 [file JCMM-23-798-s002.tif]

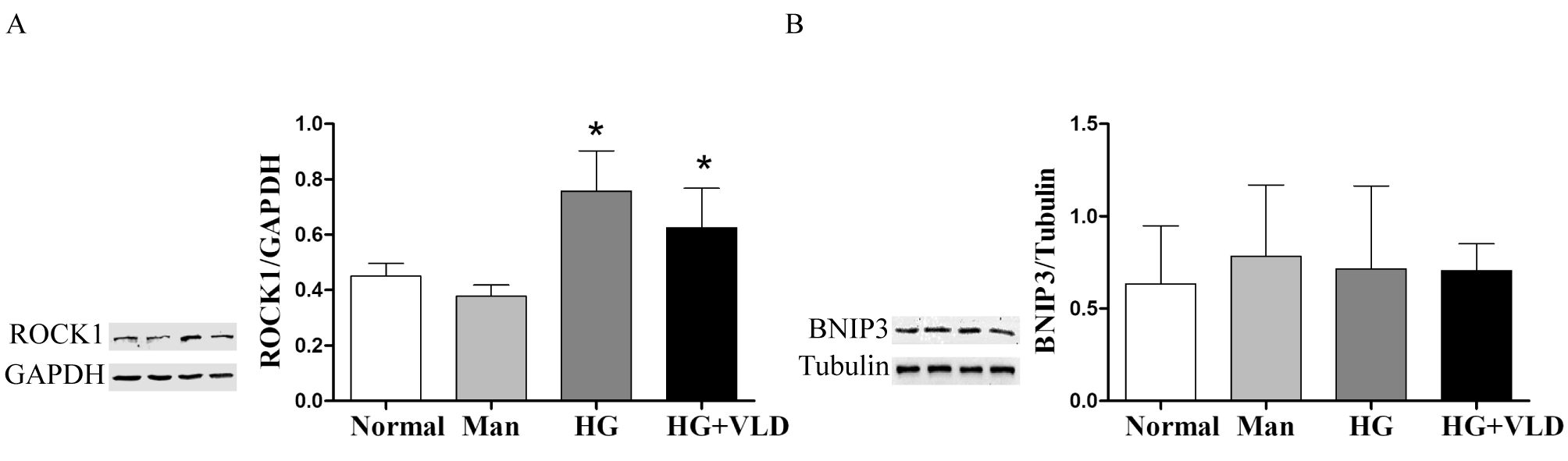

Supplement: Supplementary file 3 [file JCMM-23-798-s003.tif]
