# Supplementary material for: Vildagliptin improves high glucose‐induced endothelial mitochondrial dysfunction via inhibiting mitochondrial fission
Source: J Cell Mol Med. 2018 Nov 16;23(2):798–810. doi: 10.1111/jcmm.13975 (PMC6349192; doi:10.1111/jcmm.13975)
Supplement: Supplementary file 4 [file JCMM-23-798-s004.docx]

**Materials and Methods**

**Reagents** Primary antibodies against Drp1 (180769), MFN1 (ab57602), MFN2 (ab56889), OPA1(ab42364) and BNIP3 (ab109362) were from Abcam (Cambridge, MA, USA), and primary antibodies against pAMPK (Thr 172 ) (2535S), acetyl-CoA carboxylase (ACC, 3662S), pACC (Ser79) (3661S), pDrp1(Ser637)(4867S), and GAPDH (2118S) were purchased from Cell Signaling (Beverly, MA, USA).

**Cell culture** Human umbilical vein endothelial cells (HUVECs) purchased from American Type Culture Collection (Manassas, VA, USA) were grown in DMEM supplemented with 10% FBS at 37℃ in a 5% CO_2_ humidified atmosphere.

**Figure Legends**

FIGURE S1. Effects of VLD on the expression of fission related proteins(Drp1 and Fis1), upstream molecules and Drp1 phosphorylation level at Ser637 in HUVECs with normal glucose treated. HUVECs were treated with or without 1 µM VLD (the VLD group) for 48h, and then the levels of Drp1, Fis1, pAMPK, pACC and pDrp1 were evaluated by Western blot. A. Effects of VLD on the expression of Drp1 and Fis1. B. Effects of VLD on the proteins expression of upstream molecules and Drp1 phosphorylation level at Ser637. NS, no siginificance *vs* the normal group; VLD, vildagliptin; HUVECs, human umbilical vein endothelial cells. pAMPK, phosphorylation of AMPK at Thr172 ; pACC, phosphorylation of ACC at Ser79; pDrp1, phosphorylation of Drp1 at Ser637.

FIGURE S2. Effects of VLD on the expression of mitochondrial fusion proteins in HUVECs under high glucose condition. HUVECs were treated with 5.6 mM glucose (normal glucose group, normal group), 5.6 mM glucose plus 25 mM mannitol (Man group, osmotic pressure control), 30 mM glucose (high glucose group, HG group), or 30 mM glucose plus 1.0 µM VLD (HG+VLD group) for 48h, and the levels of MFN1, MFN2 and OPA1 were evaluated by Western blot. VLD, vildagliptin; HUVECs, human umbilical vein endothelial cells; HG, high glucose; Man, mannitol.

FIGURE S3. Effects of VLD on the expression of ROCK1and BNIP3 in HUVECs under high glucose condition. HUVECs were treated with 5.6 mM glucose (normal glucose group, normal group), 5.6 mM glucose plus 25 mM mannitol (Man group, osmotic pressure control), 30 mM glucose (high glucose group, HG group), or 30 mM glucose plus 1.0 µM VLD (HG+VLD group) for 48h, and the levels of ROCK1 and BNIP3 were evaluated by Western blot. VLD, vildagliptin; HUVECs, human umbilical vein endothelial cells; HG, high glucose; Man, mannitol. *^*^P* <0.05 *vs* the normal group
